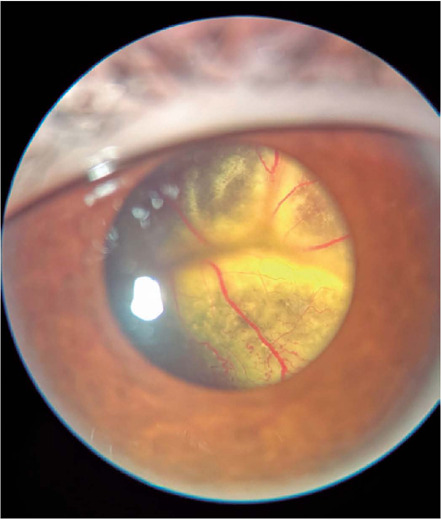# Retinal detachment in Coats’ disease

**DOI:** 10.5935/0004-2749.2024-0155

**Published:** 2024-08-30

**Authors:** Giulia Aragão, Nicole B. M. Almeida, Newton Kara Junior

**Affiliations:** 1 Ophthalmology Department, Hospital das Clínicas, Universidade de São Paulo, São Paulo, SP, Brazil

Coats’ disease is an idiopathic exudative retinopathy that is characterized by retinal telangiectasias, aneurysms, and capillary nonperfusion. It is associated with intraretinal and subretinal exudations, which frequently progress to exudative retinal detachment^(1)^. Coats’ disease is mostly unilateral and progressive and predominantly affects males during childhood. Although the average age at the time of diagnosis is 8-16 years, adult cases have also been described^(2)^. The most commonly used classification was proposed by Shields et al., and it is based on funduscopic findings. Although this classification can aid in the disease diagnosis^(1)^, in a majority of the patients, some form of ancillary testing, such as fluorescein angiography, ultrasound, computerized tomography, or magnetic resonance imaging, is required^(2)^. The most common manifestations of the disease are decreased visual acuity, strabismus and leukocoria^(3)^. The differential diagnoses of Coats’ disease include retinoblastoma, retinal vasoproliferative tumor, familial exudative retinopathy, retinal capillary hemangioblastoma, and familial retinal arterial macroaneurysm^(1)^. The aim of treatment in Coats’ disease is the ablation of abnormal retinal vasculature, preservation of vision, and prevention of disease progression to retinal detachment^(3)^. Thus, the treatment options include photocoagulation, cryotherapy, and surgery^(1)^. Antivascular endothelial growth factor or corticosteroids may also be injected intravitreally as adjuvant therapy^(1)^.

**Figure:**